# A Natural Isoquinoline Alkaloid With Antitumor Activity: Studies of the Biological Activities of Berberine

**DOI:** 10.3389/fphar.2019.00009

**Published:** 2019-02-14

**Authors:** Da Liu, Xue Meng, Donglu Wu, Zhidong Qiu, Haoming Luo

**Affiliations:** ^1^Department of Pharmacy, Changchun University of Chinese Medicine, Changchun, China; ^2^Key Laboratory of Effective Components of Traditional Chinese Medicine, Changchun University of Chinese Medicine, Changchun, China

**Keywords:** berberine, biological activities, antitumor, autophagy, epigenetic effects

## Abstract

Coptis, a traditional medicinal plant, has been used widely in the field of traditional Chinese medicine for many years. More recently, the chemical composition and bioactivity of Coptis have been studied worldwide. Berberine is a main component of Rhizoma Coptidis. Modern medicine has confirmed that berberine has pharmacological activities, such as anti-inflammatory, analgesic, antimicrobial, hypolipidemic, and blood pressure-lowering effects. Importantly, the active ingredient of berberine has clear inhibitory effects on various cancers, including colorectal cancer, lung cancer, ovarian cancer, prostate cancer, liver cancer, and cervical cancer. Cancer, ranked as one of the world’s five major incurable diseases by WHO, is a serious threat to the quality of human life. Here, we try to outline how berberine exerts antitumor effects through the regulation of different molecular pathways. In addition, the berberine-mediated regulation of epigenetic mechanisms that may be associated with the prevention of malignant tumors is described. Thus, this review provides a theoretical basis for the biological functions of berberine and its further use in the clinical treatment of cancer.

## Introduction

Natural medicine plays a very important role in novel drug discovery (Zhang et al., 2013; Zhang L. et al., 2017). In recent years, many natural products have been confirmed to play an important role in cancer prevention and therapy (Tao et al., 2015; Zhang et al., 2015, 2016; Meng et al., 2018). Coptis chinensis is a valuable Chinese medicine used commonly in China. The medicinal parts are the dried rhizome of *Coptis chinensis* Franch., *Coptis deltoidea* C.Y.Cheng, and P.K.Hsiao, or *Coptis teeta* Wall (Wang et al., 2015b). It has been reported that Coptis exerts antibacterial, immune-enhancing, anti-ulcer, hypoglycemic, detoxifying, antitumor, and other pharmacological effects (Imenshahidi and Hosseinzadeh, 2016). Coptis is mainly used for the adjuvant treatment of depression, coronary heart disease, diabetes, liver cancer, and other malignant tumors. There are several active ingredients of Coptis chinensis, such as berberine (BBR), palmatine, coptisine, jatrorrhizine, worenine, columbamine, cedarone, obakunone, obakulactone, magnoflorine, and ferulic acid; berberine is the main bioactive component of Coptis chinensis and is present at a content of 5.20–7.69%. Consequently, it has become one of the natural small-molecule drugs used commonly in the clinical setting treatment for chronic disease such like diabetes (Cicero and Baggioni, 2016; Tabeshpour et al., 2017).

**FIGURE 1:**
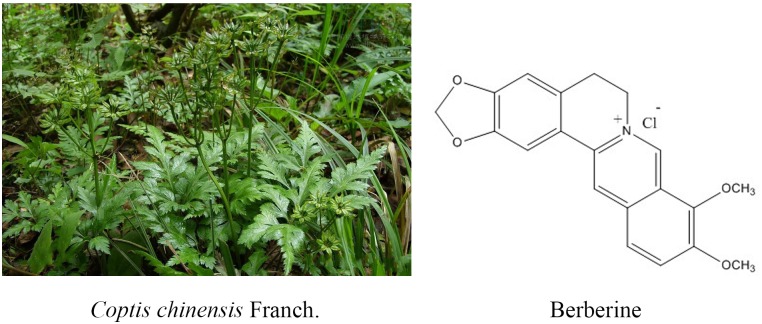
*Coptis chinensis* Franch. and chemical structure of berberine.

Berberine hydrochloride, the more commonly available salt form of berberine, is a quaternary ammonium isoquinoline alkaloid with the chemical formula C_20_H_18_ClNO_4_ (Figure 1) that forms yellow needle-like crystals (Neag et al., 2018). Berberine was originally used as a broad-spectrum antibacterial drug. Extensive research revealed a wide range of pharmacological activities, including antibacterial, anti-inflammatory, antihypertensive, hypolipidemic, and antidiarrheal effects. In addition, berberine exhibits inhibitory effects on a variety of tumors (Xu et al., 2017), such as esophageal cancer. Many studies (Kumar et al., 2015; Foroutan F. et al., 2018; Foroutan T. et al., 2018; Mirhadi et al., 2018) have confirmed that berberine affects the development of tumor cells through the inhibition of tumor cell growth and the induction of apoptosis and cell cycle arrest (Iizuka et al., 2000; Kong et al., 2004; Tang and Feng, 2009; Xue et al., 2013; Signorelli et al., 2017).

It is reported that 8.2 million people die of cancer every year globally and that this number is continuously rising; according to the American Cancer Society, cancer is the cause of more than 600,000 deaths every year in the United States, a mortality rate second only to heart disease (Khalil et al., 2016; Walker et al., 2017). Owing to the seriousness of this situation, scientific approaches to the prevention and control of cancer have become a major public health issue (Gu et al., 2015; Viegas et al., 2017).

It has long been believed that the occurrence and development of tumors are attributable to only genetic abnormalities, which include gene mutations, translocations, and chromatin insertions (Dupont et al., 2009; Li et al., 2018). However, in recent years, the emergence and progress of genome sequencing technology have led to the rapid development of epigenetics and many researchers have determined that epigenetics plays an important role in the regulation of tumors. Epigenetic changes are reversible, heritable changes in gene expression and protein function in which the genomic DNA sequence remains unchanged (Biswas and Rao, 2018). Epigenetic changes can regulate gene expression at multiple levels, for example, at the DNA level through DNA methylation, at the RNA level through non-coding RNA regulation, at the protein level through histone modification, and at the chromatin level through chromatin remodeling. The continuous presence of these mechanisms in cell division allows cells to retain their respective characteristics, respond to intrinsic cellular signals, and participate in cell evolution and adaptation to environmental changes. Many research studies have confirmed that epigenetic mechanisms are implicated in tumorigenesis through the regulation of oncogene activation and tumor suppressor gene inactivation. For example, DNA methylation can inactivate tumor suppressor genes, abnormal histone acetylation can change tumor-associated gene expression, and non-coding microRNAs can result in dysregulation of tumor suppressor genes (Blandino et al., 2014; Wong and Chim, 2015). It is of note that different epigenetic modifications in cells often interact with each other in a synergistic manner to maintain body’s homeostasis through the regulation of the expression of key genes, and that when abnormal changes occur, they may cause a variety of diseases, including tumors (Vijayaraghavalu et al., 2013). Recent evidence has suggested that epigenetic modifications may be involved in the processes tumor cells use to shape a microenvironment suitable for their own growth (Honda et al., 2006). There are a large number of active substances, such as growth factors, inflammatory factors, and proteases, in the tumor microenvironment and these participate in the various processes of tumorigenesis through their own functional properties or mediated signaling pathways (Booth and Gutierrez-Hartmann, 2015). Epigenetic modifications are involved in the regulation of the secretory processes of these biomolecules or their mediated signaling pathways (Li et al., 2018). From the perspective of the tumor development process, the regulation of epigenetic modification in the tumor microenvironment occurs at various stages of tumorigenesis, progression, and metastasis, and is one of the important tools for diversifying between tumor cells and the tumor microenvironment. That is to say, tumors may have specific epigenetic modification characteristics that may lead to changes in cell biological characteristics and malignant transformation. Therefore, an exploration the mechanism of tumor biology from the perspective of epigenetics is of great significance.

## The Biological Efficacy of Berberine

### Berberine Inhibits the Migration and Invasion of Tumor Cells

Migration and invasion are the basic characteristics of tumor cells. Therefore, it is valuable to study whether berberine can affect the migration and invasion ability of tumor cells. It is well known that E-cadherin and *N*-Cadherin proteins are closely related to cell migration and invasion. Moreover, E-cadherin is not only an important mediator that regulates cell-cell adhesion, but also an important molecule in the maintenance of the morphology and structural integrity of epithelial cells (Qi et al., 2014; Shi et al., 2017). There is a large amount of experimental evidence suggesting that berberine can inhibit the migration and invasion of tumor cells. In human lung cancer A549 cells, berberine increased the expression of E-cadherin protein in a concentration- and time-dependent manner (Li et al., 2018), and significantly downregulated the expression of *N*-cadherin; these changes inhibited invasion and metastasis. MMPs are a class of important proteins that are involved in that the degradation of the extracellular matrix barrier, which is the first step in tumor cell metastasis (Hao et al., 2017). Studies have shown that berberine inhibits the expression of MMP2 and MMP9 in a time- and concentration-dependent manner. Simultaneously, berberine also regulates the expression of MMPs through the inhibition of the transfer of p-STAT3 to the nucleus, which affects its activity. Wang X et al. found that berberine was an effective inhibitor of the invasion and migration of HCC cells. Berberine treatment of HCC cells downregulated the expression of cox-2, NF-κB, uPA, and MMP9 in a dose-dependent manner (Sengupta et al., 2017). In summary, the data strongly suggest that berberine has an important role in the regulation of cadherin- and MMP-mediated pathways, which leads to inhibitory effects on cancer migration and invasion (Table 1).

**Table 1:** Inhibitory effects of berberine on tumor migration and invasion.

Cell lines	Mechanism	Reference
Human non-small cell lung cancer (A549)	N-Cadherin↓ E-cadherin↑	Li et al., 2018
Hepatocellular carcinoma (HCC) cells	COX-2↓,NF-κB↓ UPA,MMP-9↓	Sengupta et al., 2017
Endothelial canjcer colon cells	IL-8↓, NF-κB↓ AMPK↑	Li et al., 2014
Mouse melanoma cell (B16F-10)	VEGF mRNA↓	Siveen and Kuttan, 2011; Hamsa and Kuttan, 2011
Human umbilical vein endothelial cells (HUVEC)	COX-2↓, iNOS↓, VEGF mRNA↓	Ma et al., 2008
Human endometrial cancer cell lines(AN3 CA and HEC-1-A)	COX-2↓ PEG-2↓	Wu et al., 2012

Furthermore, Jin Y. et al. (2017) showed that the effect of berberine on the metastatic potential of cancer cells may be mediated by the activation of the AMPK signaling pathway, which reduces the activity of ERK and the expression of COX-2, thereby inhibiting the adhesion, migration, and invasion of tumor cells. Moreover, berberine inhibited tumor cells through signaling pathways, including the NF-κB and AMPK pathways. Studies have demonstrated that berberine prevents tumor cells from producing IL-8 and blocks NF-κb signaling pathway, ultimately inhibiting endometrial cancer metastasis, and that colon cancer cell migration was inhibited by targeting AMPK signaling (Li et al., 2014).

Vascular endothelial growth factor, the most important angiogenic factor secreted from tumor cells, stimulates tumor neovascularization through an increase in the mitogenic and survival properties of vascular endothelial cells. Berberine not only reduces the expression of SC-M1 cells with normal oxygen content and low oxygen content. VEGF also directly inhibits the proliferation and migration of umbilical vein epithelial cells. Berberine treatment in B16F-10 melanoma cells reduced the expression of VEGF mRNA and inhibited angiogenesis. Inflammation plays an important role in tumor angiogenesis, which is mainly manifested through the activation of NF-κB to regulate VEGF, and results have shown that berberine treatment of tumor cells significantly inhibited NF-κB and ultimately decreased the expression of VEGF and IL-8 in tumor cells (Hamsa and Kuttan, 2011; Siveen and Kuttan, 2011). In addition, berberine significantly inhibited the VEGF-induced migration and invasion of human umbilical vein endothelial cells HUVEC in a dose-dependent manner, and significantly reduced the expression of COX-2, Inos, and VEGF mRNA and downregulated pro-angiogenic factors to inhibit angiogenesis (Naveen et al., 2016; Wang et al., 2018). These results indicated the critical effects of berberine on the HIF1α/VEGF pathway.

Angiogenesis plays an important role in tumor growth, as progression and metastasis are prerequisites for solid tumor growth. The angiogenic process is therefore a target for the inhibition of tumor growth and metastasis (Ma et al., 2008) Studies have shown that berberine can reduce the levels of IL-1β, IL-6, TNF, and GMCSF in the serum of tumor-inoculated animals and inhibit the elevation of NO and TNF-α, inflammatory mediators involved in angiogenesis. Wang Y et al. inferred that berberine suppressed the growth and metastasis of endometrial cancer cells via miR-101/COX2, and berberine is also known to inhibit tumors via the COX-2/PGE2 signaling pathway. The transcription of miR-101 is up-regulated by berberine through AP-1 to regulate the transcription of COX-2 in EC cells (Wu et al., 2012). The high expression of p-STAT3 in malignant tumor cells and the expression level of p-STAT3 in tumor tissues, the more obvious the proliferation and metastasis of tumor cells (Munir et al., 2000).

### Berberine Inhibits Tumor Cell Proliferation (Autophagy, Apoptosis)

Apoptosis is an ideal form of cell death in cancer therapy because it generally does not cause an inflammatory response. Thus, the induction of apoptosis is one of the various mechanisms that inhibit the growth of tumor cells (Yakata et al., 2007). It has also been reported that berberine significantly inhibited the proliferation of human prostate cancer PC3 cells (Huang et al., 2015). In recent years, studies have shown that the proliferation of renal cell cancer cells can be effectively inhibited by berberine; when a certain concentration of berberine is treated to renal cell cancer cells, the effects continue for some time. The inhibitory effect of berberine on the tumor cells gradually increased, and it was found that the effect of inhibitory effect was greatest for treatment times of up to 48 h. In addition, the total apoptotic rate of renal tumor cells detected by a double staining method showed that after treatment of renal cell cancer cell lines A498 and 786-0 with different concentrations of berberine, the rate of total apoptosis in cells gradually increased as the concentration of the drug increased (Wang et al., 2015a; Liu et al., 2017a,b, 2018).

Berberine induces apoptosis in tumor cells, mainly through upregulation of pro-apoptotic genes and downregulation of apoptosis-inhibiting genes. For example, berberine can upregulate the expression of the pro-apoptotic protein BAD in HL-60 cells and downregulate the expression of anti-apoptotic protein Bcl-2 to achieve regulation of tumor cell apoptosis. In addition, apoptosis can be induced by the mitochondrial/caspase pathway, DNA cleavage induces tumor cell apoptosis, tumor cell apoptosis is induced by inflammatory factors, and tumor cell apoptosis is induced by cyclooxygenase. For example, berberine treatment of liver cancer cells revealed that DNA fragments, caspase-3, and caspase-8 were activated, which was followed by the activation of PARP, and the release of cytochrome c to inhibit tumor metastasis (Mistry et al., 2017).

Studies have showed that berberine can regulate apoptosis-associated proteins. Caspase cleavage is a typical phenomenon in apoptosis cells. Thus, numerous reports have used the detection of this cleavage to clarify the role of berberine in the induction of apoptosis. For example, berberine decreased the expression of Bcl-2 and survivin and, conversely, increased the expression of the pro-apoptotic genes Bax and cleaved caspase-3 in a dose-dependent manner in human ovarian cancer SKOV3 cells (Su et al., 2015). Moreover, the treatment of berberine to treat human colorectal adenocarcinoma (HCT-15) cells significantly increased the expression of spliced caspase-3 and the mitochondrial apoptosis-related protein Bax, and significantly decreased the expression of Bcl-2 and survivin, finally inducing apoptosis (Agnarelli et al., 2018). Berberine inhibited the proliferation of human cervical cancer Ca Ski cells through alteration of the ratio of p53 and Bax/Bcl-2 proteins, upregulation of ROS, and enhancement of caspase-3 activity to induce apoptosis (Kalaiarasi et al., 2016). In addition, berberine induced the proliferation of BIU-87 and T24 cells through the inhibition of protein expression, the induction of G1 cell cycle arrest, and the induction of apoptosis via the caspase-3 and caspase-9 pathways (Lu et al., 2015). Agnarelli A et al. treated U343 cells and MIA PaCa-2 cells with 50 μM berberine for 48 h, and found that the activity of caspase-3 was decreased in U343 cells and increased in MIA PaCa-2 cells. Therefore, they concluded that berberine promoted the apoptosis of tumor cells (Katona et al., 2014). It has been reported that berberine induces Bax activation in human lung cancer A549 cells, enables p53 pathway-mediated cytochrome c release, and leads to the activation of caspase signaling ultimately causing apoptosis (Shi et al., 2013). The reported data also showed that berberine induced cancer cell apoptosis mainly through the regulation of the expression of caspases and Bcl-2; this results in the release of cytochrome c and the activation of the mitochondria-dependent apoptotic pathway to promote apoptosis in PC3 cells (Wang et al., 2017).

Autophagy is one type of cellular self-protection mechanism, consisting mainly of the degradation of macromolecular material and damaged organelles in cytoplasm after autophagosome formation with lysosomes. The products of degradation are used to restore cell homeostasis. There are three forms of cell autophagy: macro-autophagy, micro-autophagy, and autophagy, which are mediated by different molecular chaperones. Autophagy is involved in many of the physiological and pathological processes of cells, and there is a close relationship between autophagy and tumorigenesis. The effects of autophagy vary in different cell lines and maybe inhibitory or stimulatory. In addition, the occurrence of autophagy is regulated by various signal pathways. Recent experimental studies have shown that berberine inhibits the proliferation of colon cancer cells through the downregulation of the expression of EGFR and that it activates autophagy and apoptosis through the p38 signaling pathway to inhibit the proliferation of HCT-15 cells. Similarly, in berberine-treated HCT-15 cells, the autophagy marker proteins ATG5 and LC3 were upregulated in a time-dependent manner (Zhang L. et al., 2017), indicating that berberine induced autophagy in HCT-15 cells. These data demostrate a role of Berberin in regulating cancer cell proliferation (Tables 1, 2).

**Table 2:** Inhibitory effects of berberine on tumor cell proliferation.

Cell Lines	Mechanism	Reference
Liver cancer cells	Caspase-3↑, Caspase-8↑,PARP↑	Mistry et al., 2017
Human ovarian cancer cell (SKOV3)	Bcl-2↓ Bax↑, Cleaved-Caspase-3↑,	Su et al., 2015
Colorectal adenocarcinoma cell line (HCT-15)	EGFR↓, Bcl-2↓, Survivin↓ ATG5↑, Bax↑, LC3↑	Agnarelli et al., 2018
Human cervical cancer cell (CaSki)	p53↑, Bax/Bcl-2↑ ROS↑, Caspase-3↑	Kalaiarasi et al., 2016
Human bladder cancer cell (BIU-87, T24)	Caspase-3↑, Caspase-9↑	Lu et al., 2015
Human pancreatic carcinoma cell (MIA PaCa-2)	Caspase-3↑ P53↓	Katona et al., 2014

### Berberine Arrests Tumor Cell Cycle

Many studies have shown that low concentrations of berberine arrest human osteosarcoma U20S cells in the G1 phase through the induction of DNA double-strand breaks that activate the p53-p21 pathway. In contrast to low concentrations of berberine, high concentrations induce arrest in the G2/M phase, but do not depend on the p53-p21 pathway (Yang et al., 2015; Li et al., 2017). Other studies demonstrated that berberine significantly inhibited human ovarian cancer cells (HEY and SKOV3 cells) in a time- and dose-dependent manner. It is demonstrated that that berberine exerts a significant inhibitory effect on human gastric cancer MGC 80 3 cells in a dose-dependent manner. Using laser confocal microscopy, the nucleus condenses, and apoptotic bodies are seen, which indicate that berberine can inhibit the proliferation of MGC 80 3 cells and arrest cells in the G0/G1 phase to inhibit the proliferation of tumor cells *in vitro*.

B-cell translocation gene 2 is a transient early-response gene induced by p53. It is a member of the gene family that regulates cell proliferation and is an important bridge molecule that links p53, pRB, the cell cycle, cell proliferation, and differentiation. The current body of evidence indicates that berberine can promote the cell cycle arrest of human hepatoma HEPG2 cells in the G1 phase through the upregulation of BTG2 and the downregulation of cyclin D1, consequently inhibiting the proliferation of hepatoma cells and inducing apoptosis.

Cyclin is one of the target proteins that regulate the G1 phase. As a proto-oncogene, it is involved in the regulation of the cell cycle, and its overexpression is closely related to the occurrence and development of tumors. Berberine has a variety of effects on the cell cycle; for example, it can arrest the G2/M phase in the cell growth cycle through a reduction in the expression of cyclin B1 and increase in the expression of Wee1, which stops the tumor cells in the early stage of DNA synthesis (G1) and late DNA synthesis (G2). The induction of tumor cell apoptosis through the downregulation of cyclin E expression and upregulation of p21 expression, which causes G1 arrest in HEY and SKOV3 cells and downregulates Bcl-2 protein expression and upregulates Bax protein expression. Berberine treatment of MDA-MB-231 and MCF-7 human breast cancer cells dose-dependently caused G0/G1 arrest, which was possibly associated with a decrease in the cell cycle regulation protein cyclin B1. Furthermore, it increased the expression of CDC4 and cyclin B1 through an increase in the expression of CDC2 and caspase-3 in human hepatoma HepG2 cells, causing arrest in the S and G2/M phases, and activating the AMPK signaling pathway to induce the apoptosis of HepG2 cells (Chidambaram et al., 2012; Murthy et al., 2012; Balestrieri et al., 2018). Li et al. demonstrated that berberine regulates the PI3K-AKT and MAPK signaling pathways in PTC (the most common subtype) and ATC (the most malignant and aggressive subtype), leading to mitochondrial apoptosis, G0/G1 cell cycle arrest, increased Bax/Bcl-2, cleaved caspase-3, p21, and decreased cyclin E1, CDK2, and vimentin were verified by western blotting (Waterbeemd et al., 2013). The combination of drugs upregulated the expression of the cell cycle-dependent kinase inhibitory proteins p27 and p21, and downregulated the expression of cyclin D1, CDK2, and CDK4-cyclin.

In addition, studies have reported that berberine can bind to topoisomerase (TOP1), which hinders the synthesis of S phase cells and prevents cell proliferation.

### Effects of Berberine in Compatibility

With the identification of numerous anti-tumor drugs, research of cancer therapy has gradually shifted from a focus on monotherapy to combined therapy. More and more reports have demonstrated that berberine combined with radio-therapy or chemotherapy drugs can achieve better anti-tumor effect. For instance, berberine combined with gamma-radiation enhance the anti-cancer effects, including inducing apoptosis and ROS generation (Jung-Mu et al., 2009). Also, berberine sensitizes lung cancer cells to radiation via autophagy both *in vitro* and *in vivo* (Peng et al., 2008). Indicated an adjuvant role in radio-therapy of cancer. Another major anti-cancer therapy is chemotherapy, several novel chemotherapy drugs such like doxorubicin, rapamycin were texted combined with berberine, and showed a more effective result. It is reported that berberine sensitizes mutliple human cancer cells to the anticancer effects of doxorubicin (Tong et al., 2012). More details and drugs were summarized in Table 3, which clarified that berberine synergistic work with chemotherapy drugs in anti-tumor proliferation through inducing cell cycle arrest, apoptosis, as well as autophagy. These data have laid theoretical foundation for the combined therapy in clinic trial.

**Table 3:** Berberine combined with chemotherapy drugs.

Combined With	Cells	Mechnisms/Effect	Reference
2-deoxy-D-glucose	Human lymphoblastoid TK6 cells	BBR combined with the glucose analog 2-deoxy-D-glucose (2-dG) synergistic inducing the apoptosis of human lymphoblastoid TK6 cells	Halicka et al., 2017
5-Fluorouracil	Gastric cancer cells AGS	BBR sensitized gastric cancer cells to 5-FU, the combination shows a synergistic inhibition of surviving and STAT3 level	Pandey et al., 2015
Cinnamaldehyde	Lung carcinogenesis A549 cell	BBR combined with cinnamaldehyde prevented A549 cell substance permeability via AMPK-reduced AQP-1 expression	Meng et al., 2017
Cisplatin	Breast cancer MCF-7	BBR sensitized MCF-7 cells to cisplatin through inducing DNA breaks and caspase-3-dependent apoptosis	Zhao et al., 2016
D-limonene	Human gastric carcinoma cell line MGC803	BBR in combination with d-limonene showed synergistic anticancer effects on MGC803 cells through inducing cell-cycle arrest, ROS production, and apoptosis via the mitochondria-mediated intrinsic pathway	Zhang et al., 2014
Doxorubicin	Murine melanoma B16F10 cells	BBR combined with Doxorubicin inhibit melanoma tumor growth through casepase-3 depentdent apoptosis	Mittal et al., 2014
	Lung cancer cell lines	BBR sensitizes lung cancer cells to Doxorubicin by promoting STAT3 degradation, inhibiting doxorubicin mediated STAT3 activation.	Zhu et al., 2015
Evodiamine	Breast cancer MCF-7	BBR in combination with evodiamine inducing cell cycle arrest and apoptosis, further inhibit MCF-7 prolieration	Du et al., 2017
Hsp90 inhibitor NVP-AUY922	Colorectal cancer	BBR combined with NVP-AUY922 inhibit proliferation of colorectal cancer via mutiple pathways	Su et al., 2015
Metformin	NSCLC	BBR combined with metformin synergistic induced cell cycle arrest, as well as reduced migration and invasion of NSCLC cells	Zheng et al., 2018
Rapamycin	Human hepatoma cell SMMC7721 cells	BBR combined with rapamycin can improve HCC therapy through inhibiting the mTOR signaling pathway	Guo et al., 2014
S-allyl-cysteine (SAC)	Human liver cancer HepG2 cells	BBR combined with SAC effectively reduced Rb-phosphorylation resulting insignificant nuclear E2F presence, further inhibiting cancer cell proliferation	Sengupta et al., 2017
	DEN+CCl4 induced hepatocarcinoma	BBR in combination with SAC inhibited Akt mediated cell proliferation, and inducing PP2A/JNK mediated apoptosis.	Sengupta et al., 2014
Sorafenib	Human liver cancer SMMC-7721 and HepG2 cells	berberine combined with sorafenib inhibited the proliferation of liver cancer cells by inducing cancer cell apoptosis.	Huang Y. et al., 2018
Tamoxifen	Breast cancer MCF-7	BBR sensitized MCF-7 cells to tamoxifen via inducing the G1 phase arrest and activating apoptosis.	Wen et al., 2016
Tetrahydropalmatine	MDA-MB-231 breast cancer cells	BBR combined with tetrahydropalmatine synergistic inhibited the proliferation of MDA-MB-231	Zhao et al., 2014
TPD7	T-cell acute lymphoblastic leukemia cell	BBR combined with TPD7 induced G1 -phase cell-cycle arrest of T-cell acute lymphoblastic leukemia cell.	Ma et al., 2017

## Epigenetic Effects of Berberine on Tumors

For many years, researchers have been studying and developing drugs for cancer prevention and treatment. Chinese medicines, such as berberine, are commonly used as drugs. As an active ingredient of Coptis, berberine is inevitably closely related to the occurrence and development of tumors (Wang-Johanning et al., 2008; Coward et al., 2014; Delgado-Cruzata et al., 2015; Dkhil et al., 2015). Extensive research has led scholars to conclude that, ultimately, the antitumor effect of berberine may be related to epigenetic effects. The following is a brief description of the methods through which berberine regulates tumor cells, including migration, proliferation, and apoptosis, through epigenetic mechanisms.

### DNA Methylation

DNA methylation refers to the covalent attachment of the fifth carbon atom of cytosine on the CpG dinucleotide to the methyl group through the action of DNMT, which is modified to 5-methylcytosine. DNA methylation is a potential epigenetic mechanism involving a variety of biological processes. The DNMT family consists of three main members: DNMT1, DNMT3A, and DNMT3B. DNMT1 mainly maintains DNA methylation status and DNMT3A and DNMT3B catalyze new DNA methylations (Kalinkova et al., 2018; Li et al., 2018; Puneet et al., 2018). Human CpG exists mainly in two forms: one is dispersed in genomic DNA; the other is highly aggregated to form CpG islands, which are present in the promoter region or the first exon region of various genes. In the human genome, the CpG site is usually in an unmethylated state in the CpG islands, but in a methylated outside the CpG islands. When tumors occur, the degree of unmethylation of CpG sites outside CpG islands increases, whereas the CpG sites in CpG islands are highly methylated, causing a decrease in the overall methylation level of the genome, as well as certain gene CpG islands. Local methylation levels are abnormally elevated, leading to genomic instabilities, such as chromosomal instability, the activation of proto-oncogenes, and the silencing of tumor suppressor genes (Qing et al., 2014; Crawford et al., 2018; Lee and Gang, 2018; Sanna et al., 2018). DNA methylation abnormalities are mainly divided into the hypomethylation state of proto-oncogenes and the hypermethylation state of tumor suppressor genes. The most studied of these is the hypermethylation of tumor suppressor genes. It is of interest that berberine has been found to inhibit the expression of human DNA methyltransferases DNMT1 and DNMT3B in multiple myeloma U266 cells. For example, berberine can alter the CpG methylation of p53 DNA, affect the mRNA expression of key apoptosis-related proteins, and increases apoptosis in U266 cells, and thereby leads to cell cycle arrest. Although the hypomethylation of the p53 promoter can regulate apoptosis-related genes, such as GADD45, Bax, PMAIP1, BBC3, CCNB1, CCND3, and CCNE1. Specifically, in the p53 pathway, CDKN1A, GADD45B, Bax, PMAIP1, and BBC3 were upregulated, and CCNB1, CCND3, and CCNE1 were downregulated, which suggested that berberine activated the p53 signaling pathway through the impairment of U266 cells. In addition, results have shown that treatment of colorectal cancer cells with berberine results in a significant increase in the expression of DNMT1 and DNMT3A in the presence of TGF-β1; this hypermethylation in the promoter CpG island leads to further silencing of TSG, which results in tumor cell proliferation (Riaz et al., 2015; Asadi et al., 2018; Nardi et al., 2018).

### Histone Modification

Histones play an important role in gene expression and tumorigenesis and development. The nucleosome is the basic constituent unit of chromatin. A nucleosome is an octamer composed of histones H2A, H2B, H3, and H4 and DNA entangled on the outside of the 147 base pairs. Histones not only protect the DNA structure and genetic information, but also participate in the regulation of gene expression. The extracellular amino terminus of histones can be modified by a variety of enzymes to form specific “histone codes” that alter the “open” or “closed” state of the local chromatin structure, or determine which proteins bind to specific DNA regions. Consequently, they regulate the various functions of DNA, including transcription and damage repair. Histone acetylation is highly dynamic and is coordinated by HATs and HDACs, and occurs at the amino-specific lysine residues of histones (Stevens et al., 1984; Yan et al., 2001; Bhat et al., 2018; Georgoff et al., 2018; Liu et al., 2018; Rahnamoun et al., 2018). Histone modification is a major determinant of the epigenetic silencing of genes and the regulation of cellular processes. Histone modifications often occur at the amino terminus of histones, and modifications of various chemical groups are acceptable due to exposure to chromatin (Oliver et al., 2013; Bennetzen and Wang, 2014; Hoen and Bureau, 2015; Yamada et al., 2015; Zhang et al., 2015; Li and Zhao, 2016; Rayan et al., 2016; Jiang et al., 2018; Salimian et al., 2018; Shang et al., 2018; Yan et al., 2018; Sahebi et al., 2018; Zhang L. et al., 2018; Zhang S. et al., 2018). The most widely studied are the acetylation and methylation of lysine K on histone H3 and H4. Histone acetylation plays an important role in the epigenetic theory proposed in recent years. Histone acetylation can affect the chromatin structure in cells, and thus participate in the transcriptional regulation of genes at specific sites, playing an important role in cell growth and differentiation. With a deeper understanding of the mechanism of histone acetylation in gene transcriptional regulation, the role of HDAC inhibitors in tumor therapy has received increasing attention (Sun et al., 2017).

CBP and p300 proteins with acetylase activity are transcriptional coactivators and hematopoietic tumor suppressors. Studies have shown that berberine can upregulate the expression of CBP/P300 and SIRT3 in U266 cells, and downregulate the expression of HDAC8; however, in HL-60/ADR and KG1-α cells, CBP/P300 and SIRT3 were also upregulated, but HDAC8 did not change significantly. Histone acetylation maintains its balance through HAT and HDAC. Berberine downregulated HDAC in human lung cancer A549 cells, which resulted in decreased expression of the metalloproteinases MMP-2 and MMP-9 mRNA and protein, inhibiting cell migration and invasion (Huang et al., 2017). Simultaneously, another study has shown that berberine treatment of A549 cells significantly reduced the expression of class I, II-a, II-b, and IV mRNA, histone H3, and H4 hyperacetylation.

### MicroRNA

MicroRNAs (miRNAs) are short-chain non-coding RNAs of 19–22 nucleotides in length that bind to the 3′UTR in target mRNAs, thereby degrading or blocking the translation of target mRNAs. It plays an important role in the growth, differentiation, apoptosis, and tumor cell development. miRNAs can regulate the expression of multiple tumor-associated genes. In accordance with the function of miRNAs, they can be divided into two types: oncogenes or tumor suppressor genes (Hu et al., 2013). Not only can it act directly as a proto-oncogene or a tumor suppressor gene, but also regulate the expression of other proto-oncogenes or tumor suppressor genes. miRNAs play a central role in many cellular biology processes, and their dysregulation is a ubiquitous feature in tumors. Epigenetic effects have been shown to be a major cause of miRNA dysregulation in tumors (Hashiguchi et al., 2017). In the TGF-β1-induced colorectal cancer model, berberine significantly decreased the expression of miR-152 (targeting DNMT1), miR-429 (targeting DNMT3A), and miR-29a (targeting DNMT3A/3B), which suggested that berberine inactivates some tumor suppressor factors, including DNMT1 and DNMT3A/3B, through the regulation of the expression of the above miRNAs during colon cancer development. Furthermore, other evidence has suggested that berberine treatment of human U266 multiple myeloma cells led to the inhibition of NF-κB nuclear translocation via Set9-mediated lysine methylation, which resulted in decreased miRNA21 and Bcl-2 expression, inducing the cells to produce ROS and promoting cell apoptosis. Berberine treatment of colorectal cancer cells increased the expression of miR-200a-5p and decreased the expression of miR-429. These epigenetic regulation affected by Berberine was briefly summarized in Table 4.

**Table 4:** Epigenetic regulation of berberine on tumor.

Type of Regulation	Cell Lines	Mechanism	Reference
DNA methylation	Multiple myeloma	DNMT1↓, DNMT3B↓	Asadi et al., 2018; Riaz et al., 2015; Nardi et al., 2018
		CDKN1A↑, GADD45B↑	
		Bax↑, PMAIP1↑	
	U266 cells	CCNB1↓, CCND3↓, CCNE1↓	
Histone Modification	Human non small cell lung cancer A549	HDAC↓MMP2↓ MMP9↓	Huang et al., 2017
	U266 cells	CBP/P300↑ SIRT3↑	Sun et al., 2017
MicroRNA	Human U266 multiple myeloma cells	miRNA21, Bcl-2↓	Hashiguchi et al., 2017
	Colon cancer	miR-152↓;	Hu et al., 2013
		miR-429↓	
		miR-29↓a	

## Summary and Future Perspectives

**FIGURE 2:**
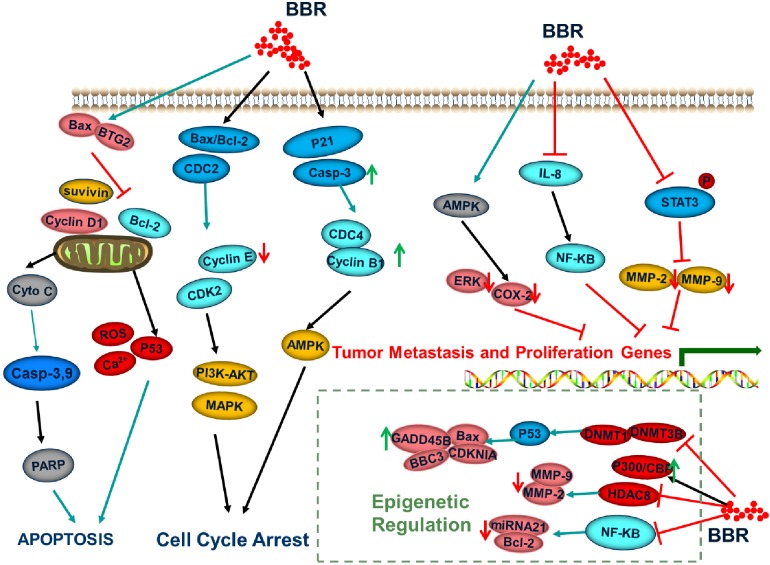
Signaling pathways regulated by berberine.

The importance of epigenetic regulation in the occurrence and development of tumors is now an established fact. An increasing body of research has been devoted to the exploration of epigenetic molecular markers for the early diagnosis, treatment, and prognosis of tumors. Simultaneously, epigenetic drugs provide a new direction for the treatment of tumors owing to the reversibility and ease of regulation of epigenetics. At present, the anticancer drugs that inhibit the proliferation of malignant tumor cells via induction of apoptosis or that regulate signal transduction are mostly multi-targeted (He et al., 2010). Berberine is a natural isoquinoline alkaloid that significantly contributed to the development of anticancer drugs (Figure 2). Given the continuous development in the field of medicine and the extension of research and development in the field of medicine, berberine has gained attention of researchers owing to the combination of multiple effects. Berberine is not irreplaceable with respect to its traditional pharmacological activities, such as antibacterial, anti-inflammatory, and antiviral effects (Huang S.X. et al., 2018). Moreover, the efficacy of the antihypertensive, antitumor, and hypolipidemic effects has also become a “hot topic” in contemporary research. Berberine regulates the molecular mechanisms that cause tumor cells through a variety of signaling pathways, confirming the potential therapeutic effects in a variety of tumor cells. However, there are few reports on the effects of berberine on the epigenetic functions of tumors. Epigenetics is also the main controlling factor of oncogenes in the development of cancer. Therefore, the application of epigenetic properties of berberine in the treatment of malignant tumors offers broad prospects for drug development. At the same time, extended research into epigenetics has provided a new strategy to understand the various characteristics of tumors, optimize the early diagnosis of tumors, and improve the prognosis of patients. In future, basic research and clinical transformations in the epigenetics of cancer will provide new strategies for the precise diagnosis and treatment of cancer.

## Author Contributions

DL contributed significantly to analysis and manuscript preparation. XM contributed significantly to manuscript preparation. DW helped to writing the manuscript. ZQ and HL contributed to the conception of the study.

## Conflict of Interest Statement

The authors declare that the research was conducted in the absence of any commercial or financial relationships that could be construed as a potential conflict of interest.

## Abbreviations

AP-1: activating protein 1
AMPK: AMP-activated protein kinase
BTG2: B-cell translocation gene 2
Bax: BCL2 associated X
BBC3: BCL2-binding component 3
CCNB: cyclin B
CCND: cyclin D
CCNE: cyclin E
CDK: cyclin-dependent kinases
CDKN1A/p21: cyclin-dependent kinase inhibitor
COX2: cyclooxygenase-2
DNMT: DNA methyltransferase
EGFR: epidermal growth factor receptor
GMCSF: granulocyte-macrophage colony-stimulating factor
GADD45: growth arrest and DNA damage-inducible 45
HCC: hepatocellular carcinoma cells
HAT: histone acetyltransferase
HDAC: histone deacetylase
HIF1α: hypoxia-induced factor α
INOS: inducible NO synthase
IL-8: interleukin-8
MMPs: matrix metalloproteinases
NSCLC: non-small cell lung cancer cells
NF-κB: nuclear factor kappa B
PARP: poly-ADP ribose polymerase
PGE2: prostaglandin E2
STAT3: signal transducer and activator of transcription 3
SIRT: sirtuins
Bcl-2: the B cell lymphoma-2
TNF: tumor necrosis factor
uPA: urokinase-type plasminogen activator
VEGF: vascular endothelial growth factor
